# Predicting symptom severity in PSTVd‐infected tomato plants using the PSTVd genome sequence

**DOI:** 10.1111/mpp.13469

**Published:** 2024-07-02

**Authors:** Jianqiang Sun, Yosuke Matsushita

**Affiliations:** ^1^ Research Center for Agricultural Information Technology National Agriculture and Food Research Organization Tsukuba Japan; ^2^ Institute of Plant Protection National Agriculture and Food Research Organization Tsukuba Japan

**Keywords:** machine learning, prediction, PSTVd, tomato, viroid, viroid‐induced symptom

## Abstract

Viroids, one of the smallest known infectious agents, induce symptoms of varying severity, ranging from latent to severe, based on the combination of viroid isolates and host plant species. Because viroids are transmissible between plant species, asymptomatic viroid‐infected plants may serve as latent sources of infection for other species that could exhibit severe symptoms, occasionally leading to agricultural and economic losses. Therefore, predicting the symptoms induced by viroids in host plants without biological experiments could remarkably enhance control measures against viroid damage. Here, we developed an algorithm using unsupervised machine learning to predict the severity of disease symptoms caused by viroids (e.g., potato spindle tuber viroid; PSTVd) in host plants (e.g., tomato). This algorithm, mimicking the RNA silencing mechanism thought to be linked to viroid pathogenicity, requires only the genome sequences of the viroids and host plants. It involves three steps: alignment of synthetic short sequences of the viroids to the host plant genome, calculation of the alignment coverage, and clustering of the viroids based on coverage using UMAP and DBSCAN. Validation through inoculation experiments confirmed the effectiveness of the algorithm in predicting the severity of disease symptoms induced by viroids. As the algorithm only requires the genome sequence data, it may be applied to any viroid and plant combination. These findings underscore a correlation between viroid pathogenicity and the genome sequences of viroid isolates and host plants, potentially aiding in the prevention of viroid outbreaks and the breeding of viroid‐resistant crops.

## Graphical abstract

The severity of symptoms induced by viroids in host plants can be predicted from genome sequences using unsupervised machine‐learning algorithms without the need for biological experiments.

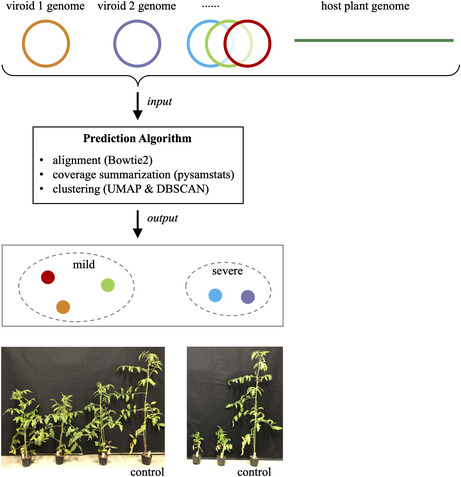

## 1 INTRODUCTION

Viroids, first identified in the 1970s, are notable for their compact structure, typically comprising approximately 240–430 nucleotides (nt) of single‐stranded, circular, non‐coding RNA (Diener, 2003; Ding, 2009; Navarro et al., 2021). Devoid of protein‐coding capabilities, viroids rely on enzymes within host plants to replicate themselves, spreading throughout the host plants via the plasmodesmata and phloem and transmitting to other individuals via vegetative propagation, physical damage (e.g., crop handling with viroid‐contaminated farming tools), pollination, or biological factors (e.g., insects and parasitic plants) (Ding, 2009; Hadidi et al., 2022).

Viroid‐infected plants may exhibit various symptoms, including stunting, leaf curling, bent leaves, chlorosis, size reduction, and deformation of flowers, fruits, and tubers (Flores et al., 2005). Symptoms range from latent (invisible) to mild, moderate, severe, or even lethal, depending on the specific combination of viroid isolates and host plant species (Hadidi et al., 2017). Because viroids are transmissible between different plant species (Verhoeven et al., 2010; Yanagisawa & Matsushita, 2017), asymptomatic viroid‐infected plants can be latent infection sources for other plant species that may exhibit severe symptoms, potentially leading to rapid and widespread transmission. Furthermore, unlike other plant diseases (e.g., powdery mildew and downy mildew) and pests (e.g., aphids and thrips), viroid infections cannot be controlled using agricultural chemicals. The sole preventive measure is the removal of infected plants, which can cause substantial economic losses globally. Consequently, predicting disease symptoms induced by viroids without biological experiments could be a rapid and effective control measure.

Potato spindle tuber viroid (PSTVd), a quintessential example of a viroid, has a broad host range. It can infect not only crop plants in the *Solanaceae* and *Asteraceae* families, such as tomatoes and potatoes, but also ornamental plants in the *Convolvulaceae*, *Liliaceae*, and *Caryophyllaceae* families (Matsushita & Tsuda, 2015). The typical length of the genomic RNA of PSTVd is approximately 359 nt; however, lengths of 341–364 nt have also been reported (Shamloul et al., 1997; Wassenegger et al., 1994). The stable secondary structure of the genomic RNA of PSTVd, resembling a rod‐like structure with double‐stranded regions flanked by loops and bulges, includes five major structural and functional domains: terminal left (TL), pathogenicity (P), central (C), variable (V), and terminal right (TR). Recent studies suggest that multiple domains, including the P domain, influence the pathogenicity of PSTVd, challenging earlier beliefs that the P domain is the sole factor (Adkar‐Purushothama & Perreault, 2020).

The pathogenicity of viroids has long been a subject of debate, yet it remains a complex and elusive topic. The link between viroid pathogenicity and RNA silencing is particularly interesting (Adkar‐Purushothama & Perreault, 2020; Flores et al., 2020). RNA silencing, also known as RNA interference, serves as a primary defence mechanism for host plants by thwarting foreign nucleic acids and regulating host gene expression. It is triggered by the presence of double‐stranded RNA (dsRNA) in host plant cells (Hamilton & Baulcombe, 1999), leading to the degradation of dsRNA into small RNAs (sRNAs) of 21–24 nt by the enzyme Dicer. Dicer‐mediated sRNAs can bind to specific host transcripts, forming partial dsRNAs that result in transcript degradation and alterations in gene expression. Previous studies have shown (i) that Dicer can degrade viroid genomes into sRNAs (Itaya et al., 2007); (ii) a considerable accumulation of viroid‐derived sRNAs (vd‐sRNAs), primarily from specific regions of the viroid genome (Adkar‐Purushothama et al., 2017; Tsushima et al., 2015); and (iii) these regions are influenced by the specific viroid isolates and host cultivars (Adkar‐Purushothama et al., 2015; Diermann et al., 2010). These observations support the hypothesis that vd‐sRNAs, resulting from RNA silencing, may target specific regions of the host genome or transcripts, disrupting gene regulation and leading to disease symptoms. Therefore, understanding the RNA silencing mechanism could offer valuable insights into predicting the symptoms of viroid‐induced diseases in host plants.

In this study, we apply the principles of the RNA silencing mechanism to develop an algorithm that predicts the severity of viroid‐induced symptoms in host plants using only the genome sequences of the viroids and their host plants. The predictive capabilities based on genome sequences alone can not only facilitate rapid and efficient assessment of potential viroid outbreaks but also provide insights that could help in breeding viroid‐resistant crops.

## 2 RESULTS

### 2.1 Variability in disease symptoms of PSTVd‐induced tomato plants

Tomato plants (*Solanum lycopersicum* ‘Rutgers’) were inoculated with 33 randomly selected PSTVd isolates. Considering the symptoms induced by KR611355 (Schnölzer et al., 1985) and AY518939 (Matoušek et al., 2012) at 2 months after inoculation as the reference for mild and severe symptoms, respectively; 15 and 11 isolates were determined to cause mild and severe symptoms, respectively (Table 1). Additionally, seven isolates that caused symptoms with severity ranging between mild and severe were determined to cause moderate symptoms. Furthermore, in a previous study using the same inoculation experimental protocol as in this study, EU879913 was determined to cause moderate symptoms, and LC523672, LC523675, and LC523676 were determined to cause severe symptoms (Matsushita et al., 2021). Taken together, we were able to collect information on the symptom severity of 37 PSTVd isolates in tomato plants.

**TABLE 1:** Symptom severity of PSTVd infection of tomato plants.

Symptom severity	PSTVd isolates (GenBank numbers)
Mild	AF483470, EF192393, EF192394, EF580923, EU879915, EU879916, JQ806338, KF418767, KR611355, KT987925, LC388852, LC388854, M25199, MG450357, Y09575
Moderate	AF454395, KF683200, KJ857496, KR611360, M88678, X17268, GQ853461
Severe	AJ634596, AY518939, AY532801, DD220185, FR851463, JX280944, U23060, X58388, X76846, X97387, Y09383

### 2.2 Expression profiles of vd‐sRNAs in PSTVd‐infected tomato plants

**FIGURE 1:**
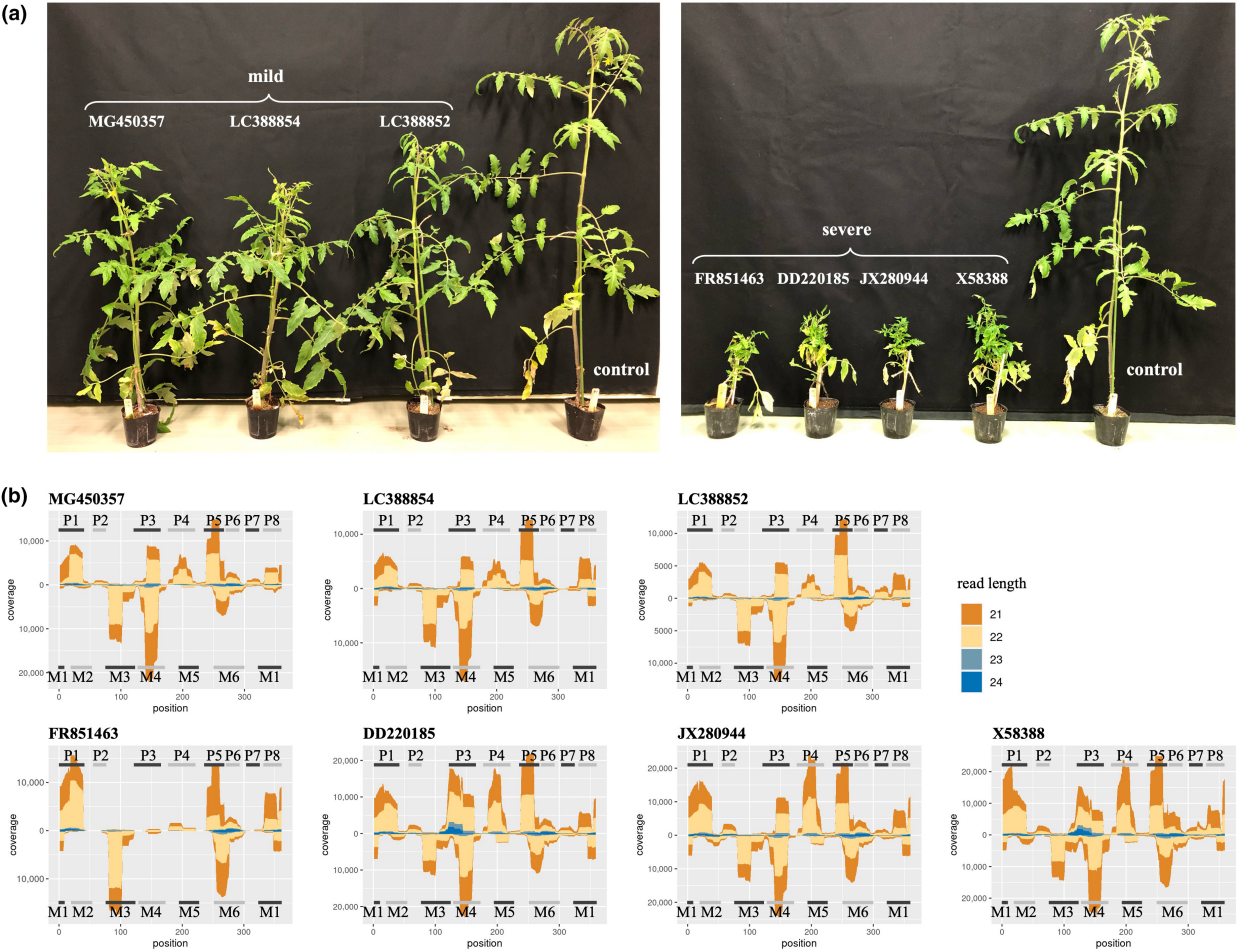
Results of the inoculation experiment and RNA‐seq analysis. (a) The infection experiment results for three and four potato spindle tuber viroid isolates clustered as mild and severe, respectively. (b) The alignment coverage of small RNA‐seq reads (21–24 nucleotides [nt]) on the genomic RNA sequence of the seven potato spindle tuber viroid isolates. The *x*‐axis represents the position of the PSTVd genomic RNA sequence, and the *y*‐axis represents the alignment coverage; the upward and downward scales represent the coverages of reads mapped on the + and − strands, respectively. The colours represent the lengths of short reads.

To explore the expression profiles of vd‐sRNAs, small RNA‐seq analysis was conducted on three (MG450357, LC388854, and LC388852) and four (FR851463, DD220185, JX280944, and X58388) randomly selected PSTVd isolates that induced mild and severe symptoms (Figure 1a), respectively. Common features among these profiles included a predominance of the aligned reads at 21 and 22 nt, whereas those at 23 and 24 nt were marginal (Figure 1b). Additionally, multiple hotspots without symmetry between the forward and reverse strands were observed. The forward strand consistently produced more vd‐sRNAs than the reverse strand.

The three isolates inducing mild symptoms (MG450357, LC388854, and LC388852) showed similar expression profiles: the forward strand featured five hotspots (P1, P3, P4, P5, and P8), with P5 showing the highest coverage. Moreover, the reverse strand had three hotspots (M3, M4, and M6), with M4 having the highest coverage among them.

Among the four isolates causing severe symptoms, DD220185, JX280944, and X58388 showed expression profiles relatively similar to those of the three isolates inducing mild symptoms. However, while the isolates inducing mild symptoms featured a single high‐coverage hotspot, P5, on the forward strand, those inducing severe symptoms displayed multiple high‐coverage hotspots, including P5. For instance, DD220185 demonstrated high coverage at hotspots P3, P4, and P5, while X58388 showed considerable coverage at P1, P4, and P5 on the forward strand. Conversely, FR851463 presented a markedly different expression profile from the other severe symptoms inducers, showing reduced hotspots—P1, P5, P8, M3, and M6 hotspots.

### 2.3 Validation of the prediction algorithm through the inoculation experiment

**FIGURE 2:**
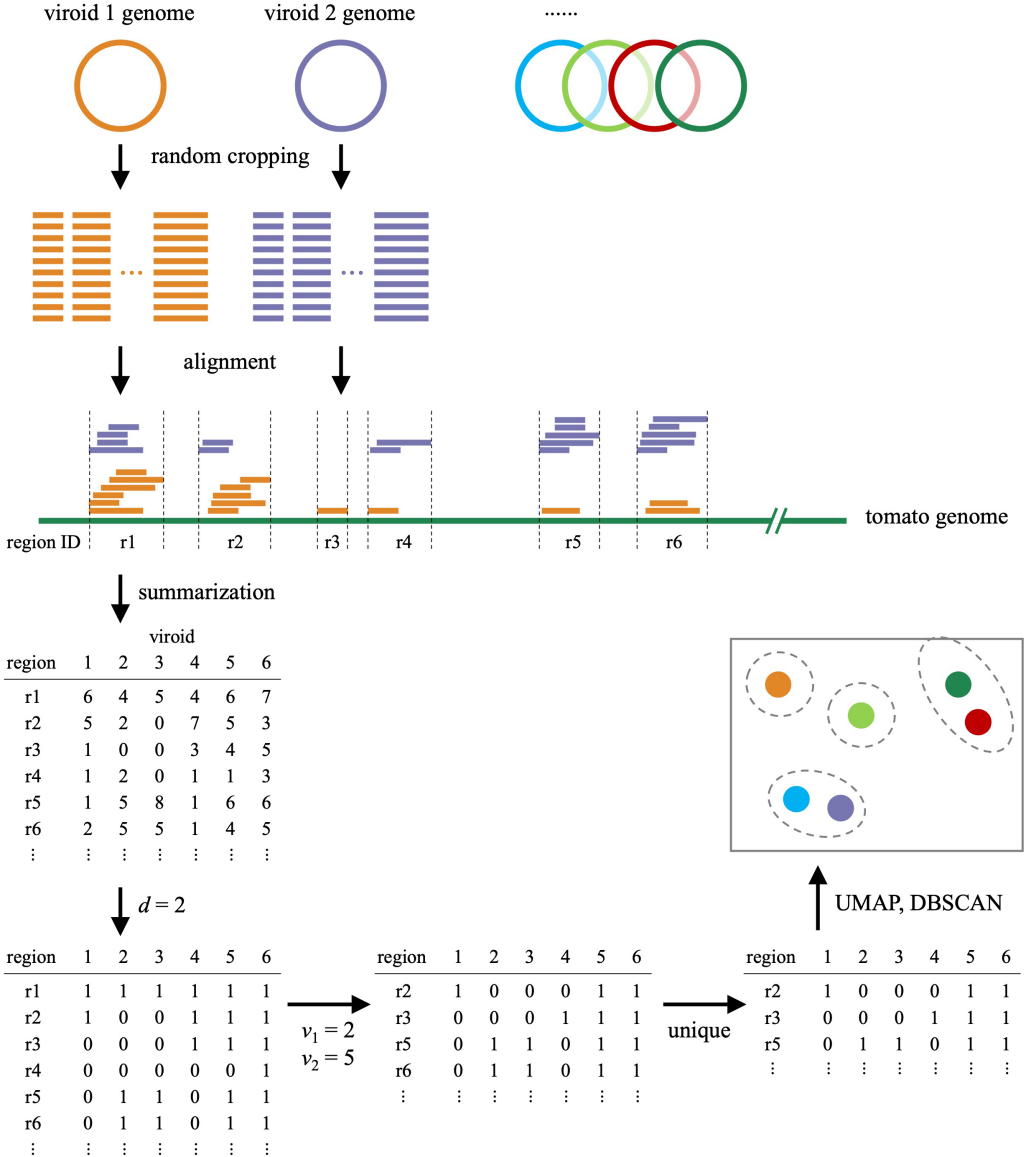
An overview of the clustering algorithm. The process of clustering potato spindle tuber viroid isolates roughly comprised three steps: alignment, alignment summarization, and clustering. In the first step, short reads (21–24 nucleotides [nt]) artificially generated from the viroid genomic RNAs were mapped on to the tomato genome sequence. Then, the mapped regions and the coverage in those regions were summarized and converted to matrix data. Finally, the matrix data were subjected to dimensionality reduction using uniform manifold approximation and projection (UMAP) and clustering using density‐based spatial clustering of applications with noise (DBSCAN).

We developed a prediction algorithm based on an unsupervised machine‐learning approach (see details in the Experimental Procedures) to predict the severity of symptoms in viroid‐infected plants. The algorithm uses the genomic RNA sequences of 306 PSTVd isolates (Data S1) and the genome sequences of the tomato plants (*S. lycopersicum* ‘Rutgers’) as inputs and assigns a cluster identification for each input PSTVd isolate as the output. This algorithm can be roughly divided into three steps: (i) aligning short reads artificially generated from viroid genomic RNAs onto the genome of the host plant; (ii) calculating the alignment coverage at each aligned region; and (iii) clustering viroid isolates according to coverage using uniform manifold approximation and projection (UMAP) (McInnes et al., 2018) and density‐based spatial clustering of applications with noise (DBSCAN) (Ester et al., 1996) (Figure 2). Although our proposed algorithm is based on an unsupervised machine‐learning approach, it requires supervised data (i.e., pairs of PSTVd isolates and their induced symptom severity) to optimize the parameters (*d*, *l*, *v*_1_, *v*_2_, *n*, *r*, *eps*, and *minPts*) and annotate the final clusters.

For this purpose, 20 of the 37 PSTVd isolates whose symptom severity was labelled were randomly selected to determine the optimal parameters of our proposed algorithm. Subsequently, we used the inoculation results of the 17 remaining PSTVd isolates to validate the clustering outcomes. Given the potential for random selection to affect model performance, we repeated the training and validation processes 100 times with varying combinations of the training and validation isolates. The average validation F1‐score was 0.85. We found that 11 cases were perfectly predictable (F1‐score = 1.0), whereas the remaining 89 simulation cases had one or two misclassifications. The isolate most likely to be misclassified was FR851463, which was predicted to induce mild symptoms in tomato plants, although the inoculation experimental results showed severe symptoms. U23060 was the next most frequently misclassified isolate and was predicted to cause mild symptoms despite causing severe symptoms in tomato plants.

**FIGURE 3:**
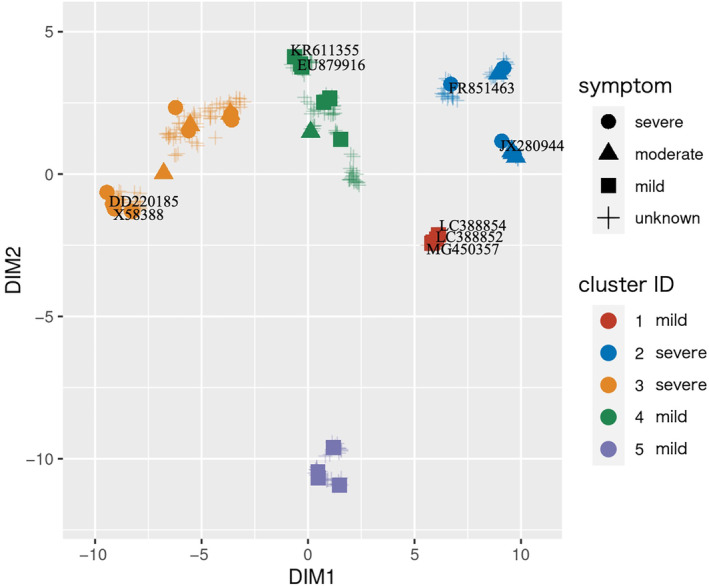
Clustering results of the potato spindle tuber viroid isolates. Clustering results of the potato spindle tuber viroid isolates based on our proposed algorithm. DIM1 and DIM2 represent the two dimensions calculated from uniform manifold approximation and projection (UMAP), and the colours represent cluster identification determined with density‐based spatial clustering of applications with noise (DBSCAN).

We then examined the 11 cases with an F1‐score of 1.0 and discovered consistent results across various training and validation subset combinations. Each case yielded six parameter combinations that produced an F1‐score of 1.0. For instance, one such combination specified the dimension of the feature vector for the PSTVd isolate (i.e., *r*) as 273, resulting in five clusters (Figure 3, Data S2). Viroids in clusters 1, 4, and 5 were predicted to cause mild symptoms in tomato plants, whereas those in clusters 2 and 3 were predicted to cause severe symptoms. Viroids causing moderate symptoms were distributed across both mild and severe clusters because these symptoms are ambiguous and can be closer to either mild or severe symptoms; therefore, they were excluded from the parameter optimization process. It is important to note that the coordinates derived from UMAP are based on a non‐linear transformation. Therefore, while the distances between points do reflect the similarities among the data points, the coordinates themselves do not carry inherent meaning. Additionally, consistent results were obtained regardless of the value of *minPts* (= 2, 4, 6, 8, and 10), indicating that the *minPts* do not considerably impact the clustering outcome.

Additionally, when comparing the expression profiles of the vd‐sRNAs with the clustering results, we found that two isolates grouped closer together in the clustering space when their vd‐sRNA expression profiles were similar (Figures 1b and 3). For example, the three isolates inducing mild symptoms (MG450357, LC388854, and LC388852) shared almost identical coordinates. Similarly, DD220185 and X58388 had approximately the same coordinates, whereas JX280944 was located far from DD220185 and X58388.

## 3 DISCUSSION

We presented an algorithm designed to predict the disease symptoms in host plants caused by viroids using only the genome sequences of the viroids and their host plants. The validation of this algorithm showed promising results: it correctly predicted the disease symptoms induced by PSTVd isolates in 11 cases and exhibited only one or two misclassifications in the remaining cases across 100 simulations. However, it should be noted that the algorithm is based on the hypothesis that vd‐sRNA affects the host genome and transcripts through RNA silencing, although no study to date provides conclusive evidence that vd‐sRNA can directly induce host transcript cleavage. Additionally, recent studies have reported that PSTVd may disrupt global alternative splicing of host transcripts (Zheng et al., 2017), alter global host genome methylation patterns (Tselika et al., 2023), and interfere with the host's endogenous factors involved in replication and transport (Ma et al., 2023). Thus, viroid pathogenicity extends beyond simple disruptions of gene regulations via RNA silencing. The fact that our algorithm, which mimics RNA silencing, failed to predict the severity of disease symptoms suggests that factors other than RNA silencing, such as those listed here, may also slightly contribute to disease development. Additionally, when dealing with sRNAs, variations between the assembled and actual genome sequences of the host plant may affect the performance of the prediction algorithm, as even a single mismatch can alter alignment outcomes. Despite these challenges, our algorithm demonstrated high efficacy in predicting the severity of symptoms induced by PSTVd isolates in tomato plants, highlighting areas for future refinement. As our understanding of the mechanisms by which viroids influence alternative splicing and methylation modification improves, we anticipate further enhancements in algorithm performance that incorporate these complex interactions.

## 4 EXPERIMENTAL PROCEDURES

### 4.1 PSTVd inoculation experiments in tomato plants

For the inoculation experiment, we randomly selected 33 out of the 307 PSTVd isolates to assess their symptom severity on tomato plants (*S. lycopersicum* ‘Rutgers’). Infectious transcripts of the PSTVd isolates were prepared via in vitro transcription using the T7 RiboMax Large Scale RNA kit (Promega), following the manufacturer's instructions. The XbaI‐linearized plasmid p94PSV (Matsushita et al., 2011), covering the full‐length sequence of the PSTVd genome and containing an 11‐nt sequence duplication (5′‐GGATCCCCGGG‐3′) downstream of the T7 promoter, was used as a template. The RNA transcripts were treated with RNase‐free DNase to remove the DNA template. The transcripts were then inoculated into tomato seedlings at the cotyledon stage (Matsushita et al., 2011). The inoculated plants were grown in a greenhouse under a temperature regime of 25°C (day) and 25°C (night) and a light/dark cycle of 16/8 h.

Two months after inoculation, the severity of symptoms in the plants was classified as mild, moderate, or severe. Symptom severity was classified as mild if the disease symptoms were similar to those of KR611355 (Schnölzer et al., 1985), whereas it was classified as severe if they were similar to those of AY518939 (Matoušek et al., 2012). Symptoms were considered mild if the plants were nearly the same height as or slightly shorter than the control individuals. Severe symptoms were noted if plants were significantly stunted when compared to the control individuals, displayed typical viroid‐induced symptoms (e.g., stunting, leaf curling, bent leaves), or if they died during the experiment. Symptoms falling between mild and severe were classified as moderate.

PSTVd‐inoculated plants were tested for viroid infection using microtissue direct reverse transcription (RT)‐PCR. Leaflets collected from the uppermost leaves of each plant were collected for over 2 months post‐inoculation for these tests. Microtissue direct RT‐PCR was conducted using primer sets P3 and P4 (Behjatnia et al., 1996; Hosokawa et al., 2006). The leaf veins of the plants were pierced three times using a white no. 3 stainless unified head‐type insect pin needle (Shiga). Samples that adhered to the needle were dipped into the RT‐PCR mixture. RT‐PCR was performed using the PrimeScript One‐Step RT‐PCR Kit Ver. 2 (TaKaRa) in accordance with the manufacturer's instructions, and the conditions were as follows: 10 min at 50°C; 2 min at 94°C; followed by 35 cycles of melting for 30 s at 94°C, annealing for 30 s at 60°C, and extension for 30 s at 72°C. The amplified PCR products were separated by agarose gel (1.5% wt/vol) electrophoresis.

### 4.2 Genome sequences of PSTVd isolates and tomato plants

We developed the algorithm using PSTVd isolates and the tomato plant as a model case. A total of 307 PSTVd isolates (Data S1) were used, with their genome sequences obtained from GenBank. The tomato plant (*S. lycopersicum* ‘Rutgers’) was used in this study. The genome sequence, version Build SL4.0, along with the corresponding annotation ITAG4.0, was downloaded from the Sol Genomics Network (Hosmani et al., 2019).

### 4.3 Analysis of small RNA‐seq data from PSTVd‐infected tomato plants

Based on the results of inoculation experiments, small RNA‐seq analysis was conducted to examine the expression profiles of vd‐sRNAs using three randomly selected (MG450357, LC388854, and LC388852) and four (FR851463, DD220185, JX280944, and X58388) PSTVd isolates that induced mild and severe symptoms, respectively. Total RNA from each sample was extracted from the uppermost leaf, c. 0.1 g in weight, for over 2 months after inoculation using the RNeasy Plant Mini kit (Qiagen) according to the method described by Yanagisawa and Matsushita (2017). RNA integrity was examined using a 2100 Bioanalyzer (Agilent Technologies). RNA sequencing libraries were constructed using the NEBNext Small RNA Library Prep Set (Illumina). All data were sequenced on the Illumina NextSeq500 platform, which generated 75‐bp single‐end reads.

The sequenced reads of the seven PSTVd isolates were pre‐processed using flexbar v. 3.5.0 (Roehr et al., 2017) for trimming adapter sequences. Only reads with lengths between 21 and 24 nt were mapped onto the genomic RNAs of the PSTVd isolates using CircSeqAlignTk (Sun et al., 2022) to summarize the expression profiles of vd‐sRNAs.

### 4.4 Algorithm for predicting the severity of disease symptoms induced by viroids

Our proposed algorithm for predicting the severity of viroid‐induced symptoms in the host plant can be roughly divided into three steps: alignment, alignment summarization, and clustering (Figure 2). The algorithm requires the genomic RNA sequences of multiple viroid isolates and the genome sequence of the host plant as inputs and produces cluster identification for each input PSTVd isolate as the output.

To generate the alignments, for each viroid, we first randomly selected a start position on the circular genomic RNA sequence of a PSTVd isolate and cropped 21, 22, 23, and 24 nt from it 10,000 times. Next, these short sequences were aligned to the tomato reference sequence using Bowtie2 v. 2.4.1 (Langmead & Salzberg, 2012) with the options ‘‐N 1 ‐L 16’.

The alignment coverage (i.e., number of reads) at each aligned region was calculated using pysamstats v. 1.1.2 (Miles, 2018) and summarized into a matrix, wherein each row indicates an aligned region, each column represents a PSTVd isolate, and each cell represents an alignment coverage of the corresponding aligned region of the corresponding isolate. The value for each cell was converted to 1 if it was larger than *d*; otherwise, it was 0. To ensure that the aligned regions correctly reflected the characteristics of the PSTVd isolates, the regions (i.e., rows of the matrix) were removed if any of the following criteria were true: (i) regions with a length less than *l* nt and (ii) regions that were common in less than *v*_1_ or more than *v*_2_ isolates. Furthermore, if multiple regions showed the same pattern, only one was retained, and the others were removed. The alignment summarization yielded a matrix with *r* rows (i.e., regions) and 307 columns (i.e., PSTVd isolates). Each column in the matrix was recognized as a feature vector (*r*‐dimensions) for the corresponding PSTVd isolate.

The dimensions of the feature vectors were reduced to two dimensions using UMAP (McInnes et al., 2018) with a parameter of *n* neighbours. Then, in a 2‐dimensional coordinate system, DBSCAN (Ester et al., 1996) with the parameters *eps* (the maximum distance between two samples to be considered as one cluster) and *minPts* (the minimum number of samples in a cluster for a point to be considered as a core point) was used to cluster the 307 PSTVd isolates.

To identify the optimal clustering parameters (e.g., *d*, *l*, *v*_1_, *v*_2_, *n*, *eps*, and *minPts*), we randomly selected 20 of the 37 PSTVd isolates used in the inoculation experiments and used the results of the inoculation experiments as a supervised label. Because moderate symptom severity may be occasionally ambiguous in comparison with mild or severe symptom severity, we used only the PSTVd isolates tagged as inducing mild and severe symptoms as supervised labels to find the best parameters using the glid search approach. After determining the best parameters, we used the remaining 17 PSTVd isolates to evaluate the clustering results. Additionally, to counteract the impact of random sampling of training and validation isolates on the clustering performance, we repeated the training and validation steps 100 times, changing the combinations of training and validation isolates.

Parameter optimization (i.e., determination of the best combination of parameters) was performed using the following combination: *d* = 0, 100, 1000, 2000, 5000, 10,000; *l* = 18, 19, 20, 21, 22, 23, 24; *v*_1_ = 0, 10, 20; *v*_2_ = 260, 280, 300, 400; *n* = 20, 40, 60, 80, 100, 120, 160, 200; *eps* = 0.1, 0.2, 0.3, 0.4, 0.5, 0.6, 0.7, 0.8, 0.9, 1.0, 1.5, 2.0, 2.5, 3.0, 3.5, 4.0, 4.5, 5.0; *minPts* = 2, 3, 5, 8, 10.

### 4.5 Calculation of validation metrics (F1‐score)

The F1‐score, a measure combining precision and recall, is calculated as the harmonic mean of these two metrics: F1‐score = 2/[(1/precision) + (1/recall)]. Precision is calculated as TP/(TP + FP), whereas recall is calculated as TP/(TP + FN), where TP, FP, and FN represent true positive (severe correctly predicted as severe), false positive (mild wrongly predicted as severe), and false negative (severe wrongly predicted as mild), respectively. The F1‐score strives to simultaneously maximize both precision and recall, thus providing a comprehensive measure of model performance.

## CONFLICT OF INTEREST STATEMENT

The authors declare no conflict of interest.

## Supporting information


**Data S1.** GenBank accession numbers of the potato spindle tuber viroid isolates. The table shows the representative and alternative GenBank accession numbers of the potato spindle tuber viroid isolates used in this study.

**Data S2.** Details of clustering results. The table shows the coordinates of the first and second dimensions of the result of uniform manifold approximation and projection (UMAP) and the predicted cluster IDs of the result of density‐based spatial clustering of applications with noise (DBSCAN). The isolates used for fitting the best parameters and validation are also shown in the table.

## Data Availability

The reference sequences of PSTVd isolates and tomato plants can be downloaded from GenBank and Sol Genomics Network, respectively. The RNA‐seq data of MG450357, LC388854, LC388852, and four isolates (FR851463, DD220185, JX280944, and X58388) were deposited in the DNA Data Bank of Japan (DDBJ) Sequence Read Archive (DRA) at www.ddbj.nig.ac.jp under the accession number DRA017371. The pre‐processed datasets and scripts for the data analyses were deposited in Zenodo at doi:10.5281/zenodo.10081178.
